# The Emerging Role of Combined Brain/Heart Magnetic Resonance Imaging for the Evaluation of Brain/Heart Interaction in Heart Failure

**DOI:** 10.3390/jcm11144009

**Published:** 2022-07-11

**Authors:** George Markousis-Mavrogenis, Michel Noutsias, Angelos G. Rigopoulos, Aikaterini Giannakopoulou, Stergios Gatzonis, Roser Maria Pons, Antigoni Papavasiliou, Vasiliki Vartela, Maria Bonou, Genovefa Kolovou, Constantina Aggeli, Aikaterini Christidi, Flora Bacopoulou, Dimitris Tousoulis, Sophie Mavrogeni

**Affiliations:** 1 Onassis Cardiac Surgery Center, 17674 Athens, Greece; georgemm32@gmail.com (G.M.-M.); vasvartela@yahoo.gr (V.V.); genovefa@kolovou.com (G.K.); 2 Department of Internal Medicine A (Division of Cardiology, Angiology, Nephrology and Intensive Medical Care), University Hospital Ruppin-Brandenburg (UKRB) of the Medical School of Brandenburg (MHB), Fehrbelliner Strasse 38, D-16816 Neuruppin, Germany; michel.noutsias@gmx.de; 3 Medical Faculty, Martin Luther-University Halle-Wittenberg, Magdeburger Strasse 8, D-06112 Halle (Saale), Germany; 4 Department of Adult Cardiology, Mitera General Hospital, Hygeia Group, 15123 Athens, Greece; angelos.rigopoulos@gmail.com (A.G.R.); sgatzon@med.uoa.gr (S.G.); 5 Aghia Sophia Children’s Hospital, 11527 Athens, Greece; aikaterinigiannakopoulou@hotmail.com (A.G.); roserpons@med.uoa.gr (R.M.P.); fbacopoulou@med.uoa.gr (F.B.); 6 Evangelismos Hospital, 10676 Athens, Greece; 7 Medical School, National and Kapodistrian University of Athens, 15772 Athens, Greece; dina.aggeli@gmail.com (C.A.); dtousoulis@hotmail.com (D.T.); 8 Iaso Children’s Hospital, 15123 Athens, Greece; theon@otenet.gr; 9 Laikon Hospital, 11527 Athens, Greece; bonou.maria@yahoo.com; 10 First Cardiac Clinic, Hippokration Hospital, 11527 Athens, Greece; 11 Euromedica Medical Clinic, 54645 Thessaloniki, Greece; catechristidi@gmail.com; 12 Clinic for Assessment of Adolescent Learning Difficulties, Center for Adolescent Medicine and UNESCO Chair in Adolescent Health Care, First Department of Pediatrics, Medical School, National and Kapodistrian University of Athens, 11527 Athens, Greece

**Keywords:** brain magnetic resonance imaging, cardiac magnetic resonance imaging, brain lesions, cardiovascular, neuro-psychiatric symptoms, cognitive dysfunction, depression, arrhythmia, heart failure, MRI

## Abstract

Heart failure (HF) patients frequently develop brain deficits that lead to cognitive dysfunction (CD), which may ultimately also affect survival. There is an important interaction between brain and heart that becomes crucial for survival in patients with HF. Our aim was to review the brain/heart interactions in HF and discuss the emerging role of combined brain/heart magnetic resonance imaging (MRI) evaluation. A scoping review of published literature was conducted in the PubMed EMBASE (OVID), Web of Science, Scopus and PsycInfo databases. Keywords for searches included heart failure, brain lesion, brain, cognitive, cognitive dysfunction, magnetic resonance imaging cardiovascular magnetic resonance imaging electroencephalogram, positron emission tomography and echocardiography. CD testing, the most commonly used diagnostic approach, can identify neither subclinical cases nor the pathophysiologic background of CD. A combined brain/heart MRI has the capability of diagnosing brain/heart lesions at an early stage and potentially facilitates treatment. Additionally, valuable information about edema, fibrosis and cardiac remodeling, provided with the use of cardiovascular magnetic resonance, can improve HF risk stratification and treatment modification. However, availability, familiarity with this modality and cost should be taken under consideration before final conclusions can be drawn. Abnormal CD testing in HF patients is a strong motivating factor for applying a combined brain/heart MRI to identify early brain/heart lesions and modify risk stratification accordingly.

## 1. Introduction

Heart failure (HF) is a rapidly increasing “epidemic” in the Western World. The improved survival of patients with coronary artery disease (CAD) and nonischemic cardiomyopathies (NICM) has significantly contributed to the rise in prevalence of HF [1]. Long-term outcome, quality of life and health care costs depend not only on HF, but also on the severity of other organs’ involvement, including the brain [1].

There is increasing evidence that HF patients frequently develop brain deficits (BD), as the disease evolves [2,3,4,5]. HF management requires a high degree of comprehension and adherence to treatment recommendations. Therefore, such deficits play a crucial role in disease control. In stable HF outpatients, lower left ventricular ejection fraction (LVEF) and brain deficits in cognitive domains predicted 1-year mortality risk [3]. Currently, information regarding the prevalence, type and severity of cognitive impairment in HF patients is limited. However, there is a great spectrum of brain findings in patients with either reduced (HFrEF) or preserved ejection fraction (HFpEF). These findings can be also found in both severely symptomatic and stable HF patients. This interaction is further supported by improvement in both organs when HF improves [6]. The aim of this review is to describe the interaction between brain/heart in HF and emphasize the role of imaging in the early diagnosis of brain/heart involvement.

## 2. Materials and Methods

A review of the literature published in English was conducted in the PubMed EMBASE (OVID), Web of Science, Scopus and PsycInfo databases. Keywords for searches included heart failure, brain lesion, brain, cognitive, cognitive dysfunction, magnetic resonance imaging, cardiovascular magnetic resonance imaging, electroencephalogram, positron emission tomography and echocardiography. The purpose for conducting this scoping review was to identify the available evidence in the field of brain–heart interaction in heart failure. We did not wish to ask single or precise questions regarding this field. In contrast, we were more interested in the identification of certain findings in relevant studies, and this is the reason for choosing a scoping review.

## 3. Results

### 3.1. Brain Anatomy and Function in HF

The critically attained threshold of cerebral hypoperfusion (‘CATCH’) theory supports the fact that aging in conjunction with vascular risk factors leads to chronic cerebral hypoperfusion and increased risk of Alzheimer’s disease (AD) [7]. Reduced myocardial contractility, commonly found in HF, leads to a decreased forward flow with concurrent decline in brain perfusion. Cerebral blood flow (CBF) reduction is further compounded by the negative action of other co-morbidities, such as hypertension, diabetes, sleep apnea and depression [8]. In HF patients, there is evidence of reduced CBF to bilateral hippocampus, parahippocampal gyrus and right posterior cingulate cortex [9,10], regions usually associated with AD. Furthermore, HF patients have up to 31% reduction in resting CBF compared with age-matched healthy controls [11]. Finally, patients with mild-to-moderate HF had reduced blood flow velocity of the middle cerebral artery, compared to healthy controls (47.3 versus 56.1 cm/s, respectively) [6]. However, the reasons of brain hypoperfusion in HF include not only the low cardiac output, due to HF, but also the compromised cerebral autoregulation [7]. Carbon dioxide levels were fluctuated in patients with either acute or chronic HF and were inversely related to left ventricular end-diastolic pressures, leading to constriction/dilatation of central nervous system (CNS) blood vessels [8]. Additionally, cerebrovascular reactivity, measured by the response of cerebral vasculature to high levels of carbon dioxide, becomes abnormal. Using transcranial Doppler to estimate CBF velocities, it was demonstrated that, whereas HF patients had baseline flow velocities comparable to normal controls, their response to the hypercapneic state, which produces vasodilation and increased flow, was decreased. Furthermore, alterations in cardiac hemodynamics, irrespective of cardiovascular risk factors and co-morbidities, are linked to reduced brain function. Interventions to maintain cardiac function in old age might have implications for preservation of brain function; therefore, physicians in charge of HF treatment should also take under consideration patients’ brain status and cognitive function. This is in agreement with recent recommendations by the AHA that cardiovascular risk factors lead to vascular cognitive impairment [9]. Finally, Heart Transplantation leads to a significant improvement in CBF, which is usually accompanied by improved cognitive performance in heart transplant patients [10].

The mental disturbances associated with HF include attention and learning deficits, memory loss, cognitive dysfunction (CD) and, to a lesser degree, language impairment and reduced visual–spatial performance [11,12]. Furthermore, HF patients have reduced cognitive function compared with matched controls [4,13,14], which remains reduced after adjustment for age, socioeconomic status and education [14,15]. Although HFrEF patients experienced more pronounced executive dysfunction and attention deficits, HFpEF patients had delayed recall and reduced abstraction abilities [16]. CD predicted poor self-care in HF patients [15], and these patients are less likely to follow their medical regimens [17,18]. Therefore, CD is a risk factor for HF decompensation, increased readmissions and mortality [18].

The evaluation of brain anatomy showed that both gray matter (GM) and white matter (WM) alterations are common in HF [19,20] and can be either diffuse or, most commonly, localized, leading to specific brain dysfunction [14,19,20,21,22,23,24]. These changes were not limited only to severely decompensated HF, but they were also seen in stable HF, with subtle CD that was detectable only through specific cognition tests [14].

### 3.2. Pathophysiology of Brain Dysfunction in Heart Failure

#### 3.2.1. The Role of Reduced CBF

Although CBF has been considered as the main causative factor of brain lesions in HF, reduced CBF cannot serve as the only explanation for cortical GM loss, where the vasculature is rich. In contrast, cardiovascular risk burden is the main cause for this [12]. HF and ischemic heart disease [IHD] patients have a similar pattern of GM loss compared with individuals with no heart disease, proving that these structural changes are related to common risk factors [23]. Furthermore, despite the similar pattern of brain injury, recent data showed that HF patients suffered GM loss in specific regions that was much more extensive than that observed in either IHD patients or healthy controls [12]. In addition, white matter hyperintensities [WMH] remained significantly more common in HF patients, even after correction for age, IHD and CAD risk factors [19]. Therefore, it seems that, although there is a strong association between reduced CBF, increased CVD risk burden and high prevalence of brain injury found in HF, brain damage cannot be explained exclusively by these factors.

#### 3.2.2. The Neurohormonal Axis

The neurohormonal axis in HF has a role in the interaction between HF, cognition and structural brain changes. Cortisol, a stress-related hormone, can influence cognitive function. Cortisol levels were found to be increased in the saliva of healthy volunteers with poor results in cognitive stress tests [25]. Furthermore, patients treated with cortisol performed worse in specific cognitive tests, compared with those treated with the placebo [26]. Although the results of these trials show that transient high levels of cortisol can directly impair the cognitive function, other studies showed that prolonged exposure to high cortisol levels can cause atrophy of specific brain regions, due to decreased neurogenesis [27]. In this context, significantly higher levels of cortisol were found in HF patients who experienced depression and CD, but not in those free from these symptoms [28], suggesting that cortisol levels in HF might influence the development of CD.

#### 3.2.3. The Inflammatory Axis

HF is a situation of increased inflammation and immune response that is usually triggered by myocardial injury [29]. In relevant trials [30], high levels of interleukin (IL)-6 and tumor necrosis factor-alpha were found in HF patients with CD and depression, but not in HF patients free from these symptoms. Additionally, in other chronic inflammatory states, such as rheumatoid arthritis, higher levels of circulating cytokines were related to significantly worse cognitive functions [31]. Notably, IL-6 receptors were found to reside specifically in areas such as the hippocampus and cerebral cortex and can trigger an intracellular cascade, resulting in subsequent neuronal loss [32].

#### 3.2.4. The Nutritional Deficiency

Keith et al. [33] showed that approximately one-third of hospitalized HF patients present thiamine deficiency. Additionally, thiamine deficiency was suspected in only 20% of patients with histologically proven Wernicke-Korsakoff brain changes [34] and the same might be hypothesized in HF. However, conclusive data regarding the role of thiamine deficiency in brain involvement of HF patients are currently missing.

#### 3.2.5. The Role of Depression

A complex interaction exists between HF, CD and depression. It was found that depression is associated with CD [35] and anatomic brain changes [36] and is also related to higher levels of inflammatory [37] and neurohormonal [38] biomarkers that are also prevalent in HF patients. Furthermore, an improvement in CD was noted in patients who were medically treated for their depression [39]. However, further studies are needed to fully clarify the role of depression in CD presented in HF.

#### 3.2.6. The Role of Atrial Fibrillation (AF)

There is evidence that AF is associated with a higher risk of cognitive impairment and dementia, with or without a history of clinical stroke. AF increases the risk of clinical stroke by four- to five-fold, and patients with a clinical history of stroke are at increased risk of developing dementia. However, AF is also associated with cognitive dysfunction, ranging from mild impairment to overt dementia, independently of clinical stroke as well as multiple shared risk factors. It is also well established that AF and cognitive impairment share common risk factors, including advanced age, diabetes, hypertension, sleep apnoea and chronic heart failure. Moreover, a significant increase of 34% was found in the risk of cognitive impairment in patients with AF in the absence of clinical stroke, even after adjustment for shared risk factors [40].

#### 3.2.7. The Role of Myocardial Infarction (MI)

The recently identified association between unrecognized MI and cerebral infarction suggests that unrecognized MI may be a novel risk factor for cardiac embolism and cerebral infarction [41].

#### 3.2.8. The Role of Heart Failure (HF)

HF is linked to an increased risk of thrombosis, leading to sudden death, stroke, systemic thrombo-embolism and/or venous thrombo-embolism. There is the risk of stroke, possibly silent, in patients with HF, even in the absence of atrial fibrillation, which may lead to cognitive dysfuncion in patients with atrial fibrillation.

In HF patients with reduced left ventricular ejection fraction who are in sinus rhythm, there is no evidence of an overall benefit of vitamin K antagonists (e.g., warfarin) on mortality, with risk of major bleeding. In contrast, risk factors associated with increased risk of thrombo-embolic events should be identified and the decision about the use of anticoagulation should be individualized. New oral anticoagulants that offer a different risk-benefit profile compared with warfarin may be an interesting alternative, but this would need to be confirmed in clinical trials [42].

### 3.3. Brain Imaging in Heart Failure

Neuroimaging includes the application of various modalities to directly or indirectly image the structure, function or pharmacology of the brain [19] and falls into two broad categories:

1. Structural imaging

This deals with brain structure and the diagnosis of large-scale intracranial disease, such as tumor or injury.

2. Functional imaging

This is used to diagnose metabolic diseases and fine lesions such as those found in AD as well as for neurological and cognitive-psychology research. Functional imaging allows the direct visualization of brain information processing through the “lights up” of the involved area.

The commonest methods to evaluate the brain include:

- Electroencephalography (EEG)

EEG is used to show brain activity in certain mental states, such as alertness or drowsiness. It is useful in the diagnosis of seizures and other medical problems involving an overabundance or lack of activity in certain parts of the brain [43].

- Positron Emission Tomography (PET)

Positron emission tomography (PET) scan measures the glucose levels in the brain to illustrate where neural firing is present and is based on the fact that active neurons use glucose as fuel. During the scan, a tracer attached to radioactive isotopes is injected into the blood, and when parts of the brain become active, blood containing the tracer is sent to deliver oxygen. This creates visible spots, which are collected by detectors and used to create images of the brain, while the patient performs a particular task. However, PET can detect only generalized areas of brain activity and not specific locations and is very expensive. A study assessing the heart–brain axis with cardiac and brain ^18^F-FDG PET/CT imaging in HF patients showed that the global and regional brain metabolic activity was significantly associated with the extent of hibernated myocardium (HM) and cardiac function [44].

- Magnetic Resonance Imaging (MRI)

MRI and functional magnetic resonance imaging (fMRI) are the most commonly used modalities in neuropsychology. MRI uses strong magnetic fields to align spinning atomic nuclei (usually hydrogen protons) within body tissues, then disturbs the axis of rotation of these nuclei and observes the radiofrequency signal generated as the nuclei return to their baseline status. Through this process, MRI creates images of the brain structure. It is noninvasive and can be used safely in patients with MRI compatible devices, cardiac valves and coronary artery stents. The most important disadvantage is that the patient has to stay still for long periods of time in a noisy, narrow space until the imaging has been accomplished.

It has been found that the CD, demonstrated in HF patients, is related to gray matter density (GMD) loss in the anterior cingulate, lateral and medial frontal cortex, regions that play an important role in strategic thinking [41]. Furthermore, despite the similar pattern of brain injury, recent data showed that HF patients had GM loss in specific regions that was much more extensive than that observed in either CAD patients or healthy controls [45]. In addition, Vogels et al. [18] showed that HF patients free from stroke, dementia or depression had a higher prevalence of WMH on brain MRI. Although WMH were previously considered the result of aging or increased cardiovascular risk burden [22,23], they remained significantly more prevalent in HF patients, even after correction for age, IHD and its risk factors [21]. Cardiac dysfunction contributes independently to the development of cerebral MRI abnormalities in patients with HF. Age and low LVEF are the principal predictors of WMH in patients with HF and in cardiac controls [22]. Furthermore, MRI brain scans, performed in dilated cardiomyopathy patients, showed significant structural brain changes, compared with normal controls, even though patients with IHD risk factors were specifically excluded from the study [24]. Finally, diminished GMD was found in wide brain regions, including the whole fronto-median cortex as well as hippocampus and precuneus, that might promote CD development [18]. This reduced GMD was correlated with decreased LVEF and increased NTproBNP [19].

fMRI measures both structure and functional activity of the brain through computer elaboration of multiple images. More specifically, fMRI measures signal changes in the brain that are due to changes in neural activity. During an fMRI scan, a patient can perform mental tasks and the area of action can be detected through blood flow from one part of the brain to another by taking pictures less than a second apart and showing where the brain “lights up”. For example, when a person processes visual information, blood rushes to the back of the brain, which is where the occipital lobe is located. fMRIs can show when things happen, how brain areas change according to this experience and which brain areas are working together [46]. Furthermore, reduced cardiac performance leads to decreased efficiency in task related brain areas and performance on a verbal working memory (VWM) task in elderly patients with CVD [47].

The brain MRI evaluation of patients with HF proved that brain dysfunction is associated with cardiac remodeling, leading to grey matter reduction in the hippocampus and the primary motor cortex, which may be involved in depressive symptoms and reduced daily activity in HF patients [48]. Furthermore, resting car diac index is associated with risk for incident dementia and Alzheimer disease (AD), including individuals free of CVD and atrial fibrillation. Therefore, even individuals with a subtle reduction in cardiac index should be evaluated for accelerated dementia and/or AD [49].

### 3.4. Cardiac Imaging in Heart Failure

Echocardiography and cardiac magnetic resonance imaging can both evaluate biventricular and atrial function. However, CMR is the modality that can characterize cardiac tissue with high accuracy/reproducibility and without the use of ionizing radiation [44].

- Echocardiography (ECHO)

Although all imaging modalities can provide a reliable estimation of ejection fraction, it is the versatility of echocardiography that makes it unique in the assessment of volumes, diastolic function, right ventricular function, hemodynamics and valvular regurgitation. The early detection of HF has been facilitated by the assessment of global longitudinal strain, which is also useful in later HF for the assessment of left ventricular (LV) dyssynchrony. The use of echocardiography has been associated with favorable outcomes, probably on the basis of facilitation of appropriate treatment. Currently, the guidelines emphasize that no single modality can answer all pathophysiologic queries in HF. However, due to high availability, low cost and great versatility, ECHO is an indispensable tool in the evaluation of HF [50]. Furthermore, echocardiography can identify atrial myopathy, which is characterized by alterations of left atrial function/size, has been recognized as a possible mechanism for ischemic stroke independently of the presence of atrial fibrillation. Impaired left atrial function may be a risk factor associated with dementia, as recently demonstrated by an exploratory analysis of a US community-based cohort [51,52].

- Cardiovascular Magnetic Resonance (CMR)

CMR is the cornerstone in the evaluation of HF because it provides comprehensive information about all clinical queries regarding pathophysiology and management of HF. Currently, CMR is regarded as the gold standard in evaluation of ventricular/atrial volumes, wall motion and systolic function of both ventricles. Due to its unique capability of performing tissue characterization, it offers incremental diagnostic and prognostic information and is considered the “sine qua non” modality in the assessment of HF [53]. Specifically, the evaluation of late gadolinium enhanced images (LGE) is considered as the gold standard for assessment of replacement fibrosis. However, lack of availability, long imaging and processing time and high cost still do not allow its wide use in clinical practice [53].

### 3.5. Role of the Proximal Aorta as a Coupling Device between Heart and Brain Perfusion

Aortic stiffness is a key mechanism of CVD risk. The proximal aorta serves as a coupling device between cardiovascular and brain function. Aging and accelerated stiffening of the proximal aorta cause increased microvascular brain pulsatility, which accelerates the development of cerebral small vessel disease.

MRI is ideal for assessing the function of the proximal aorta and clarifying the stages of brain lesions, which may contribute to early memory loss and mild CD, finally leading to dementia [54].

## 4. Discussion

### Combined Brain/Heart MRI in Heart Failure: Luxury or Real Clinical Need?

To assess CD, several standardized measures of cognitive function (CF) have been used and include the Montreal Cognitive Assessment (MoCA), the Mini-Mental State Exam (MMSE) and the Mini-Cog. All three tests measure mental functions through a series of questions and/or simple routine tasks. Although the cognitive testing cannot show the specific cause of impairment, it can assess if the patient needs further evaluation [32]. The main limitation in the evaluation of CF is the lack of robust evidence to support all available screening tests. Additionally, they have a relatively high rate of intra-subject variability that reduces their ability to identify mild deficits or preclinical disease [55]. Furthermore, there is no ideal test for any type of CD, resulting in the development of many specialized tests for various types of CD [55]. Finally, CF testing is unable to provide specific information about the neural structures responsible for any dysfunction identified. For example, although it appears that white matter function such as processing speed, attention and visual-spatial processing are particularly affected by diabetes, localization of this dysfunction to white or gray matter is not possible using the tests available to assess neurocognition [47]. A comparison of combined brain/heart MRI versus other imaging modalities is presented in Table 1.

**Figure 1:**
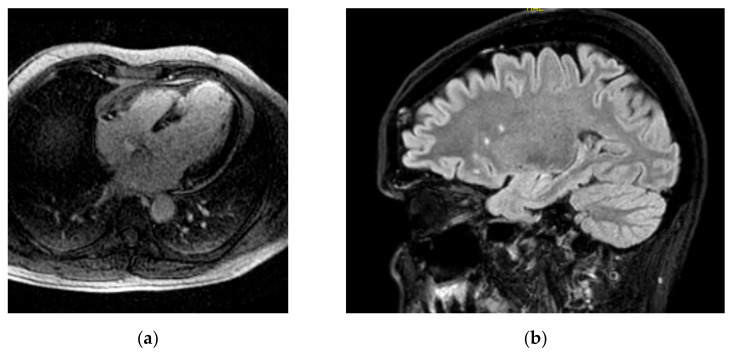
Combined brain/heart MRI images from a patient with CD and HF (**a**) inversion recovery image showing extensive myocardial LGE due to myocardial infarction; (**b**) brain FLAIR image showing WMH in the same patient.

Combined brain/heart MRI images from a patient with cognitive dysfunction and heart failure are presented in Figure 1.

At the moment, there is no data supporting a combined brain/heart evaluation in HF, except one study in autoimmune rheumatic diseases with cardiac symptoms, published by our group [56]. However, it has been documented that HF patients exhibit CD of attention and memory, and medial temporal lobe atrophy (MTA) seems to be responsible [57]. Furthermore, in stable HF, lower LVEF and brain deficits predicted 1-year mortality risk [3].

Currently, the interest of cardiologists is focused on cardiac remodeling and other events, such as inflammation/fibrosis taking place in the myocardium. However, the brain function is another important predictor of HF patients’ survival, irrespective of cardiac status, found not only in severe, but also in subtle HF [17,18]. In this context, criteria about the need of a combined brain/heart MRI should be established. Since it is not feasible to perform this combined exam in all HF patients, we should select those HF patients in whom we expect to have the maximum benefit. According to our experience, in this target group should be included:

1. HF patients with subtle CD;
2. HF patients with rapidly progressive CD;
3. Patients with rapidly progressive HF, even if they have not clinically overt CD;
4. Patients under evaluation before any change in cardiac and/or neurologic medication;
5. Patients under evaluation before any interventional or surgical treatment for structural heart disease.

In contrast to CF testing, a combined magnetic resonance imaging of brain/heart can reveal early pathophysiologic changes that are potentially clinical silent but may affect CF seriously. It is clear that this approach can be of great value in the detection of CD pathophysiology and potentially facilitates therapeutic interventions. However, availability, doctors’ familiarity with this modality and high cost should be taken under serious consideration. Finally, multicenter studies regarding the cost/benefit ratio in HF are needed before final conclusions will be drawn.

## 5. Conclusions

There is increasing evidence that HF patients frequently develop BDs that can potentially lead to CD, an important factor independently influencing their survival. CD testing is the most commonly used diagnostic approach. However, it can identify neither subclinical cases nor the pathophysiology behind the CD. A combined brain/heart MRI has the ability to diagnose these patients at an early stage and potentially influence their treatment. Additionally, valuable information about edema, fibrosis and cardiac remodeling, provided by using CMR, can improve HF patients’ risk stratification and treatment modification. However, availability and cost/benefit ratio should be also counted before this approach will be included in the routine evaluation of HF patients.

**Take home messages extracted by the scoping review**

The analysis of all available literature proved that:

1. There is a great interaction between brain and heart;
2. Any kind of cardiac pathology either subclinical or overt may influence the brain function;
3. Cognitive tests cannot identify subclinical brain lesions;
4. Early treatment of cardiac disease may prevent the development of brain lesions;
5. The prognosis of HF is dependent on brain function.

**Future research directions motivated by the scoping review**

1. A combined brain–heart MRI evaluation can be a criterion of brain–heart involvement during HF;
2. Artificial intelligence can provide more accurate and objective assessment of brain-heart MRI imaging;
3. A combination of cognitive testing and brain–heart MRI will help the categorization of HF patients in different groups of severity and facilitate treatment individualization.

## Figures and Tables

**Table 1:** Comparison of various imaging modalities for the evaluation of brain/heart interaction in heart failure.

Modalities	Brain Imaging	Cardiac Imaging	Cost	Availability	Radiation
Echocardiography	−	+	+	++++	−
PET	+	+	++++	+	+++
Combined brain/heart MRI	+	+	+++	+++	−

## Data Availability

Not applicable.
